# Effects of Aflatoxin on Liver and Protective Effectiveness of Esterified Glucomannan in Merino Rams

**DOI:** 10.1100/2012/462925

**Published:** 2012-12-13

**Authors:** Fatma Colakoglu, Hasan Hüseyin Donmez

**Affiliations:** Department of Histology and Embryology, Faculty of Veterinary Medicine, Selçuk University, 42075 Konya, Turkey

## Abstract

The effects of total aflatoxin (AF) given orally on liver in Merino rams were studied. In addition, this study was conducted in order to evaluate the efficacy of an esterified glucomannan (EG) for protection against aflatoxicosis. One-year-old 32 Merino rams were divided into four equal groups. The control group (C) was fed with the commercial feed. The AF group was fed with commercial feed plus 250 **μ**g/day of total AF. The EG group was fed with commercial feed plus 2 g/day of EG. The AF + EG group was fed with commercial feed plus 250 **μ**g/day of total AF and 2 g/day of EG. After feeding period, tissue samples were taken from the liver in order to perform histological analyses. Vacuolar degeneration with small and large droplets and hydropic degeneration in hepatocytes were observed in the AF group. The ceroid pigmentation was observed in macrophages in groups or one by one. It was observed that the fat rate in hepatocytes was 2.6% in the C group, 35.5% in the AF group, 2.9% in the EG group, and 9.6% in the AF + EG group. In conclusion, the adverse effects caused by aflatoxicosis on the liver could be ameliorated by adding EG to the ration.

## 1. Introduction

Mycotoxins are secondary metabolites of microscopic filamentous molds that have adverse effects on humans, animals, and crops that result in illnesses and economic losses [1]. Aflatoxins (AFs) are groups of structurally related mycotoxins produced as food-borne metabolites by toxigenic strains of *Aspergillus flavus* and *A. parasiticus* [2]. They are produced on cereal grains during growth, harvest, storage, or transportation [3]. It is well known that AFs are hepatotoxic, hepatocarcinogenic, teratogenic, mutagenic and immunosuppressive [4]. The AFs do not equally affect all animals. The environmental stress, sex, age, and breed differences play a significant role in toxicities. Some are more resistant, such as sheep and cattle, compared with swine, chickens, turkeys, and ducklings which are more susceptible [5]. The liver is the target organ following the ingestion of the toxin. High doses of AFs cause severe hepatocellular necrosis. On the other hand, prolonged low dosage leads to liver enlargements [6]. 

Prevention of feed and feedstuffs from possible mould growth and AF contamination is very important [7]. Practcial and cost-effective methods for detoxification of AF containing feed and feedstuff are in great demand [8]. Since the early 1990s, the adsorbent-based several studies have been performed to detoxify AF in contaminated food and foodstuffs and to minimize the deleterious effects of AF [4]. An approach to the problem has been the usage of nonnutritive and inert adsorbents in the diet to bind AF and reduce the absorption of AF from the gastrointestinal tract [9]. The nonnutritive clays such as aluminosilicates, zeolites, bentonites, and clinoptilolite were preferred by the researchers [4]. In the recent years, researchers suggested that the best approach for decontamination would be biological degradation [10]. Live yeast (*Saccharomyces cerevisiae*), initially used as a performance promoter in the early 1990s, was found to have beneficial effects on aflatoxicosis [11]. Esterified glucomannan (EG) showed considerably high binding ability (80–97%) with AF [8], and it has been preferred for detoxification of AF in poultry animals.

The aim of this study, the effects of total AF given orally on liver in Merino rams, was studied. In addition, this study was conducted in order to evaluate the efficacy of an EG for protection against aflatoxicosis.

## 2. Materials and Methods 

### 2.1. Animals and Diet

Approval for the present study was obtained from the Animal Ethics Committee of the Faculty of Veterinary Medicine of the Selçuk University (2008/061). The study was created from the TUBITAK-TOVAG project entitled “Effects of Aflatoxin on Semen Quality, Testicular Histology, and Hyaluronidase Enzyme Activity, and Protective Effectiveness of Esterified Glucomannan in Ram.”

Thirty-two Merino rams were approximately purchased 1-year old (12–14 months old). Animals were examined for general health. Antiparasitic ivermectin injection (Avromec-F, 1 mL/50 kg) and oxfendazole (Okzan-F, 1 tablet/50 kg) were performed. In addition, enterotoxemia (Pluritoxiven-8, 1 mL) and smallpox vaccines were performed. For adaptation to the environment and the new feeding implementation, it was applied the training program 15-days before starting the study. Individually weighted rams were divided into four equal groups. Experimental feeding was continued throughout the ninety-two days. The duration of treatment (92 days) was based on a possible cumulative toxicity and the duration of spermatogenesis and spermiogenesis in rams. The rams were fed a commercial food (Table 1). Water and alfalfa were given *ad libitum*. AF and EG that were mixed with 250 g commercial feed were given to animals before morning feeding and then morning feeding was continued. 

### 2.2. Experimental Design

The experimental design consisted of four dietary treatments. The control group (C) was fed with the commercial feed. The AF group was fed with commercial feed plus 250 *μ*g/day of total AF. The EG group was fed with commercial feed P 2 g/day of EG. The AF + EG group was fed with commercial feed plus 250 *μ*g/day of total AF and 2 g/day of EG. The AF and EG doses which were given to animals throughout the study were calculated by pharmacologists.

### 2.3. Aflatoxin

The AF was produced (in the Department of Pharmacology and Toxicology, Faculty of Veterinary Medicine, Selçuk University, Konya, Turkey) from *Aspergillus parasiticus* NRLL 2999 culture (USDA, Agricultural Research Service, Peoria, IL, USA) via fermentation of rice by the method of Shotwell et al. [12] with minor modifications by Demet et al. [13]. Fermented rice was sterilized in autoclave, dried at 70°C, and ground to a fine powder. According to the method reported by Vicam [14], extraction and cleaning of AF in fermented rice was used in immunoaffinity column (Down Test; Vicam). The amount of AF measured by high performance liquid chromatography (HPLC) according to the method reported by Stroka et al. [15]. The amount of total AF in the fermented rice was found 73.96 ppm. The AF within the rice consisted of 84.15% AFB_1_, 6.29% AFB_2_, 9.13% AFG_1_, and 4.25% AFG_2_ (rate of return method 97.4%; sensitivity 0.4 ppb). 

### 2.4. Collection and Processing of Tissue Samples

At the end of the 92th day, liver tissue samples were taken from rams after scarification and were fixed in 10% neutralized buffered formaldehyde, embedded in paraffin wax, and then stained with Crosman's modification of trichrome stain in order to determine the histological structure [16]. In addition, in order to determine fat rate in hepatocytes, liver samples were determined along 16 hours in formol-calcium solution, at +4°C, and dark. Sections (12 *μ*m) were taken from cryostat, and these were stained with Sudan Black B stain [17]. Fat rate in hepatocytes was determined with the method reported by Gaal et al. [18].

### 2.5. Statistical Analysis

The obtained results (ratio of fatty liver) were statistically analyzed using Duncan's multiple range test in SPSS software (version 17; SPSS Inc., Chicago, IL, USA). The level of significance was *P* < 0.05.

## 3. Results

**Figure 1:**
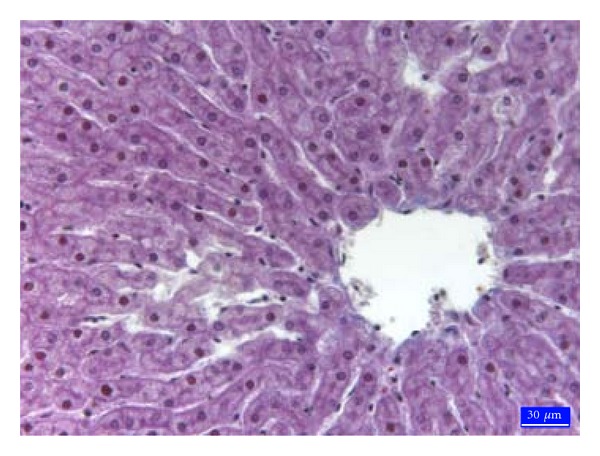
Normal histological appearance of the liver in the control group, 3rd region, trichrome staining.

**Figure 2:**
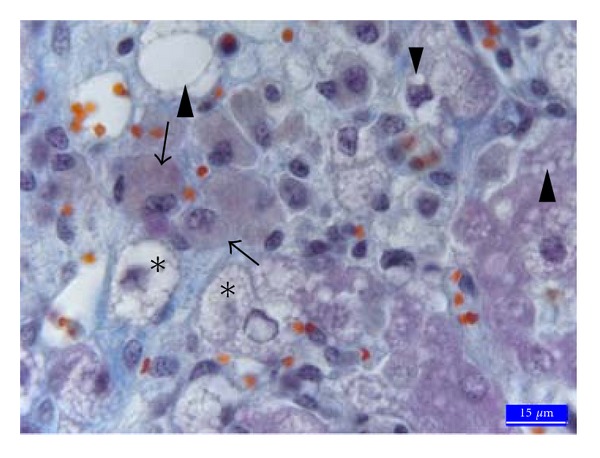
Liver histology from an animal contaminated with AF (250 *μ*g/day of total AF, AF group). Macrophages containing ceroid pigment (arrows), severe vacuolar degeneration in hepatocytes (arrowheads), and hydropic degeneration (stars) in the AF group, 3rd region, trichrome staining.

**Figure 3:**
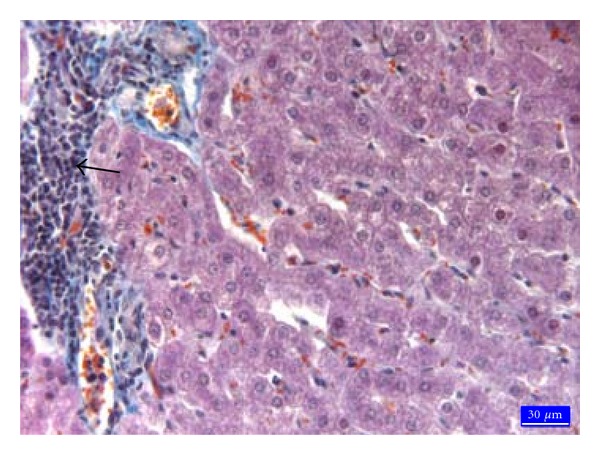
Mononuclear cell infiltration in portal areas in the AF + EG group (arrows), trichrome staining.

Histopathologically, no specific lesion was observed in liver tissues from the C (Figure 1) and EG groups. In the AF group specific lesions were seen in liver. Vacuolar degeneration with small and large droplets (Figure 2) and hydropic degeneration (Figure 2) in hepatocytes, local hyperemia, marked sinusoidal contraction, and a few hepatocytes with pyknotic nuclei in lobules were noticed. Furthermore, the ceroid pigmentation (Figure 2) was observed in macrophages and local hemorrhage areas around central vein. In the AF + EG group was seen a mononuclear cell infiltration in portal areas (Figure 3). Specific lesions like ceroid pigmentation and vacuolar degeneration in this group were not seen. It was found that fat rate in hepatocytes (Table 2) was 2.6% in the C group, 35.5% in the AF group, 9.6% in the AF + EG group and 2.9% in the EG group.

## 4. Discussion

The liver is considered the main target organ for aflatoxicosis [10]. Lakkawar et al. [6] reported that liver was the most affected organ in rabbits fed an AFB_1_ contaminated diet. The effects of AFs on histopathological changes are directly correlated with the concentration of AF and the duration of the exposure [19]. The vacuolar hepatocellular change in animals that were fed 1.6 ppm AFB_1_ diet continuously throughout 40 weeks was more than animals that were fed diets that contained 0, 0.01, 0.04, 0.4, or 1.6 ppm AFB_1_, using an intermittent dosing regimen, 4 weeks on and 4 weeks off AFB_1_ [20]. In this study, it was observed that 250 *μ*g/day AF caused significant histopathological changes in liver in 92 days.

Histopathological findings that were caused by AFs in liver were reported as enlarged nuclei, nuclear inclusions, and swollen hepatocytes [19]. Enlarged hepatocytes can arise from stored metabolism products as a result of shown activities when these cells perform metabolic activities [21], and the AF metabolites arising from the effects of some of the enzymes which react with hepatocyte DNA are thought to be caused by mutations in the nucleus [22]. Yıldırım et al. [10] saw that some hepatocytes nuclei were pyknotic and there was nucleus loss in some hepatocytes in the AF group. In this study, we also found a similar finding. A study of Ozen et al. [7] found apparently a dose-dependent mild to severe vacuolar degeneration with irregularly shaped vacuoles in hepatocytes; most of the hepatocytes also contained sharply edged vacuoles that were a sign of fatty accumulation. Kıran et al. [23] reported that hepatic lesions in broilers of the AF-treated group were characterized as diffuse and severe hydropic degeneration and periportal fibrosis. In this study, we also observed vacuolar and hydropic degeneration similar to previous studies [7, 23]. It was reported that vacuolar degeneration could be due to impaired lipid transport rather than increased lipid biosynthesis [24].

By histological examination of livers in the AF group, Lakkawar et al. [6] observed vascular congestion (up to 30 days), areas of coagulative necrosis around the central veins, and engorged portal areas (from the 40th day onwards) in rabbits. Abdel-Wahhab et al. [25] reported vascular dilation, congestion, and moderate hemorrhages in rats. Hemorrhages in animals of the AF group have also been reported in other studies [26, 27]. Although Salim et al. [28] reported dilated sinusoids depending on the hepatocytes necrosis as a result of aflatoxicosis, we observed sinusoidal contraction depending on the enlargement and swelling of hepatocytes. This finding was in agreement with that reported by Uopasai et al. [29]. Hyperemia was noted where there can be seen sinusoids. Dilation of arterioles causes hyperemia due to increased blood to the tissue depending on inflammation [30]. 

Some researchers have reported cellular disorganization characterized by the accumulation of pigments [19]. In cat's liver which has primary hepatic lipidosis, Gul et al. [31] have seen ceroid pigmentation which is Periodic Acid Schiff (PAS) positive, freely or shaped yellow large droplets in cytoplasm of hepatocyte/Kupffer cells, and more intense in the periportal areas of the liver. In the AF group, we also found hepatocytes and Kupffer's cells which were more intense around the central vein showing ceroid pigmentation. This station is a result of impaired lipid metabolism and severity steatosis [32]. 

AFs affect primarily the cellular immunity process in most animal species [4]. In the AF-exposed chickens, Yıldırım et al. [10] have declared mild mononuclear cell infiltration in the liver's portal areas. In this study, we also observed a similar finding in the AF + EG group. Small inflammatory cell infiltrates composed of lymphocytes, plasma cells, mononuclear cells, and few segmented neutrophils occur to respond to degenerate vacuolated hepatocytes [20]. 

In this study, histopathological findings obtained from the AF + EG group are close to the C group. This result has shown that EG is an important adsorbent in decreasing the detrimental effects of AFs. This finding was in agreement with the previous reports [8, 10, 19]. 

AF causes impaired lipid transport [24]. The data obtained from this study have also shown that AFs cause fatty liver in rams as in other animals [33]. Furthermore, it has been shown EG decreased significantly that fat rate in hepatocytes and the detrimental effects of AF. 

## 5. Conclusion

In conclusion, the adverse effects AF impaired on histological structure of the liver that caus fatty liver could be ameliorated by adding EG to the ration. We conclude EG is an agent which can be used successfully to prevent aflatoxicosis.

## Figures and Tables

**Table 1:** Composition of the commercial feed.

Dry matter	88%
Crude protein	12%
Crude cellulous	12%
Crude ash	9%
Insoluble ash in HCL	1.0%
Ca	0.6–1.6%
P	0.4%
Na	0.1–0.4%
NaCl	1.0%
Metabolic energy	2750 kcal/kg
Vit A	7000 IU-kg
Vit D3	700 IU-kg
Vit E	25 mg/kg

**Table 2:** Fat rate in hepatocytes (%).

Groups (*n* = 8)	C	AF	EG	AF+EG
Fat rate (%)	2.63 ± 0.21^c^	35.50 ± 0.65^a^	2.94 ± 0.25^c^	9.63 ± 0.98^b^

C: control, AF: aflatoxin, EG: glucomannan, AF + EG: aflatoxin + glucomannan.

^a,b,c^are significantly different (*P* < 0.05).
